# Wireless whispering-gallery-mode sensor for thermal sensing and aerial mapping

**DOI:** 10.1038/s41377-018-0063-4

**Published:** 2018-09-12

**Authors:** Xiangyi Xu, Weijian Chen, Guangming Zhao, Yihang Li, Chenyang Lu, Lan Yang

**Affiliations:** 1 Department of Electrical and Systems Engineering, Washington University, St. Louis, MO 63130 USA; 2 Department of Computer Science and Engineering, Washington University, St. Louis, MO 63130 USA

## Abstract

The Internet of Things (IoT)^1,2^ employs a large number of spatially distributed wireless sensors to monitor physical environments, e.g., temperature, humidity, and air pressure, and has many applications, including environmental monitoring^3^, health care monitoring^4^, smart cities^5^, and precision agriculture. A wireless sensor can collect, analyze, and transmit measurements of its environment^1,2^. Currently, wireless sensors used in the IoT are predominately based on electronic devices that may suffer from electromagnetic interference in many circumstances. Being immune to the electromagnetic interference, optical sensors provide a significant advantage in harsh environments^6^. Furthermore, by introducing optical resonance to enhance light–matter interactions, optical sensors based on resonators exhibit small footprints, extreme sensitivity, and versatile functionalities^7,8^, which can significantly enhance the capability and flexibility of wireless sensors. Here we provide the first demonstration of a wireless photonic sensor node based on a whispering-gallery-mode (WGM) optical resonator, in which light propagates along the circular rim of such a structure like a sphere, a disk, or a toroid by continuous total internal reflection. The sensor node is controlled via a customized iOS app. Its performance was studied in two practical scenarios: (1) real-time measurement of the air temperature over 12 h and (2) aerial mapping of the temperature distribution using a sensor node mounted on an unmanned drone. Our work demonstrates the capability of WGM optical sensors in practical applications and may pave the way for the large-scale deployment of WGM sensors in the IoT.

High-quality WGM optical resonators that confine light in a small volume via total internal reflection can significantly enhance the light–matter interactions, which have benefited a number of applications including microlasers^9,10^, opto-mechanics^11,12^, and non-Hermitian optics^13–15^. When subject to environmental changes, WGM resonators will experience changes in their spectral properties, e.g., frequency shift/splitting and linewidth broadening. Based on such mechanisms, WGM resonators have been demonstrated in various sensing applications, including thermal sensors^16^, humidity sensors^17^, magnetometers^18^, nanoparticle/biomolecule detection^19^, and atomic ion detection^20^. Successful laboratory demonstrations have encouraged and advanced the practical applications of WGM sensors. However, the system must address two open challenges to fully realize the power of the resonator technology for practical applications: (1) the stability of the photonic resonator and its coupler, such as a fiber-taper waveguide, and (2) the miniaturization of bulky laboratory measurement systems. Several pioneering works in these areas have been demonstrated recently, e.g., the WGM optical gyroscope^21^ and the phone-sized WGM sensing system^22^, which integrated a single WGM sensor together with its coupler, laser, photodetector, and associated control components into a portable device. The potential of WGM sensors can be further improved through integration with wireless interfaces as parts of an IoT system.

In this work, we demonstrated a wireless WGM sensor node that can be integrated into the IoT. We also developed a customized iOS app for remote system control, and collection and analysis of sensing signals. Through this app, the spectral properties of the WGM sensors can be monitored in real time. As the key elements in wireless sensor networks (WSNs)^23–25^, sensor nodes should have the capability to collect sensing signals, perform signal analysis, and communicate with other sensor nodes or the gateway sensor node. The architecture of our wireless WGM sensor node is shown in Fig. 1a, consisting of a sensing module, a microcontroller, a Wi-Fi unit, and its power supply^22^.

**Fig. 1:**
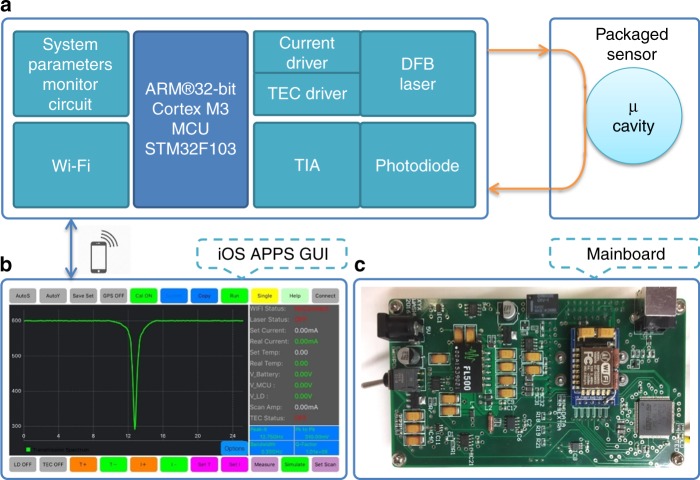
Wireless WGM sensing system. **a** Architecture of the wireless WGM sensing system. The light from a tunable single-mode distributed Bragg reflector (DBR) laser is used to probe a packaged whispering-gallery-mode sensor. The light coupled out of the sensor is sent to a photodetector with a transmission amplifier (TIA). The ARM Cortex-M3 processor is responsible for controlling peripherals including the laser current drive, thermo-electric cooler (TEC) controller, monitoring circuit, and Wi-Fi unit. The sensing system is remotely controlled by an iOS app in a smartphone via the Wi-Fi unit. **b** Screenshot of the customized iOS app for wireless control of the sensing system. The system parameters, e.g., current and temperature, can be monitored and adjusted in real time. The transmission spectrum of the packaged sensor can also be acquired and analyzed in real time. **c** Photograph of the mainboard, which integrates all the electronic components, is shown in (**a**). The size of the mainboard is ~124 mm × 67 mm

In the sensing module, a tunable single-mode distributed Bragg reflector (DBR) laser is used to probe a packaged WGM sensor;^26,27^ the output from the sensor is received by a photodiode detector. The operation of the DBR laser is controlled via its current driver and thermo-electric cooler (TEC). A transimpedance amplifier (TIA) circuit is associated with the photodiode detector and converts the photodiode current into a voltage output with the proper gain. An ARM Cortex-M3 processor (STMicroelectronics, Switzerland) serves as the microcontroller, with the main functions of laser control (e.g., voltage, current, and temperature) and acquisition of the transmission spectra of the WGM sensor. The communication between the sensor node and smartphones is via the Wi-Fi unit, which helps to transmit the sensing signals and receive commands from the customized app. By connecting the Wi-Fi unit to the internet, worldwide, real-time control of this system can be realized. We also include a system monitor circuit in the mainboard that monitors the key parameters, such as the voltages of the power supply and the microcontroller and the voltage, current, and temperature of the laser diode. A full-sized view of the mainboard is provided in Fig. 1c.

The sensor used in this system is a packaged WGM microtoroid resonator. It is fabricated using a UV curable low-index polymer to package a microtoroid together with its fiber-taper waveguide^24,27^. Such a packaged WGM sensor has a high-quality factor and long-term stability. Light from the DBR laser with a central wavelength of 976 nm and a linewidth of 10 MHz is sent into the packaged WGM sensor and then is received by the photodetector. The frequency of the laser light can be tuned by adjusting the laser current and TEC temperature with tuning coefficients of 0.002 nm/mA and 0.07 nm/°C, respectively. By applying a saw-tooth wave with an amplitude of 40 mA to the laser diode around a fixed central current, the frequency of the laser light is linearly scanned to obtain the transmission spectrum of the WGM sensor.

The interface of the customized iOS app is shown in Fig. 1b. The app can monitor the key parameters of the system in real time and remotely control the mainboard, such as setting the laser diode current and temperature and tuning the laser frequency; it can also receive the transmission spectra of the WGM sensor through the Wi-Fi unit, with a waveform update rate of 50 frames per second. Additionally, through the integrated mathematical algorithm, the app can perform real-time analyses, such as measuring the resonance frequency, the linewidth, and the quality factor. Detailed information of this iOS app, as well as a step-by-step guide, are provided in the supplementary material.

We first characterized the spectral properties of the packaged WGM sensor using the app. The transmission spectrum is shown in Fig. 2a, with a frequency span of 450 GHz. Multiple resonance modes with different resonance frequencies, quality factors, and polarizations were observed. A resonance mode with a higher-quality factor will help to resolve a smaller frequency shift, subsequently improving the sensing performance. The transmission spectrum of a high-quality mode together with a Lorentzian fit is given in Fig. 2b, with a quality factor of ~4.2 × 10^5^. To verify the stability of the whole system, the time trace of the linewidth of a resonance mode was recorded for 15 min. An average linewidth of 3.15 GHz with a standard deviation of 0.03 GHz was observed (Fig. 2c).

**Fig. 2:**
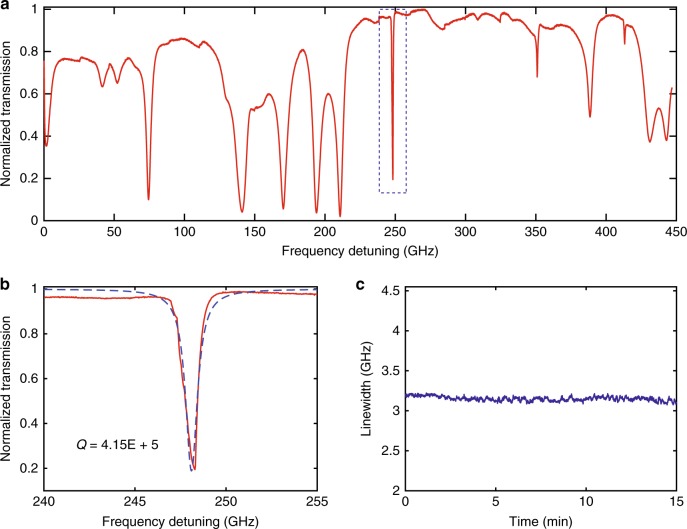
Characterization of the wireless sensing system. **a** Transmission spectrum of a packaged WGM sensor with frequency span of 450 GHz. Multiple resonance modes with different linewidths and polarizations can be obtained for sensing applications. **b** Transmission spectrum of a resonance mode (marked by a dashed box in (**a**)) with a Lorentzian fit. The quality factor is ~4.2 × 10^5^. **c** Time trace of the linewidth of a resonance mode in the wireless sensing system

With this wireless sensing system, we performed a 12-h real-time measurement of air temperature on June 18, 2017 in St. Louis, MO, USA. The whole system was mounted on the outside wall of a building. The packaged WGM sensor was in full contact with the surrounding air and was protected from direct irradiance by sunlight. The optical fibers connecting the packaged sensor with the mainboard were carefully mounted to avoid polarization variations during the measurement. The variation of the resonance frequency induced by the air temperature change was monitored via the customized app. For comparison, we also mounted a commercial thermometer together with the packaged sensor. Through the 12-h measurement, we acquired a plot of the frequency shift of the selected resonance mode. As shown in Fig. 3, the resonance frequency shift of the packaged WGM sensor matches well with the results from the commercial thermometer and has a linear dependence on the temperature change (Fig. 3 inset). The small deviation of the two curves mainly comes from the instability of the laser frequency. The deviation can be minimized by optimizing the circuit design of the laser current driver and TEC controller.

**Fig. 3:**
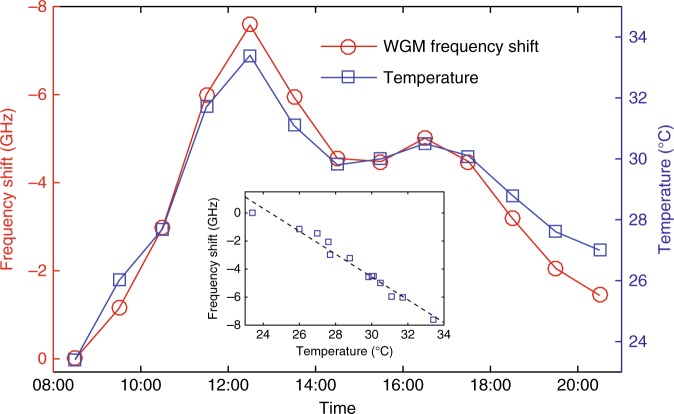
Air temperature measurement. The wireless sensing system was deployed outdoors to monitor the variation of the air temperature from 8:30 AM to 8:30 PM on June 18th, 2017 in St. Louis, MO, USA. The red circles denote the frequency shift of the selected resonance mode versus time, and the blue squares are the measurements of the temperature change by a commercial thermometer. The inset shows a linear dependence of the resonance frequency shift on the temperature change, where the blue squares denote experimental measurements and the black dashed curve is a linear fit of the experimental results

Introducing mobility to wireless sensor nodes can improve the capability and flexibility of WSNs and help meet the needs of certain scenarios with complex dynamic changes^28^. Here we used an unmanned drone to carry the whole system to measure the temperature distribution in a selected area of a city park in St. Louis on May 14, 2017 (see Fig. 4a). A commercial thermometer with a bluetooth connection was mounted along with the packaged WGM sensor for comparison. The flight path of the unmanned drone is shown in Fig. 4b, with the starting and ending locations marked. When the drone flew from one measurement location to the other, the resonance frequency of the WGM sensor shifted due to the temperature variations. The variation of the resonance frequency is shown in Fig. 4b, where the temperature gradient can be clearly seen. The measurements match well with the results from the commercial thermometer (Fig. 4c). A video demo is provided in the supplementary material, where the drone carrying the whole system flew from one location with a higher temperature (in the sunlight) to another one with a lower temperature (in the shade). A resonance frequency shift can be clearly observed using the customized mobile app.

**Fig. 4:**
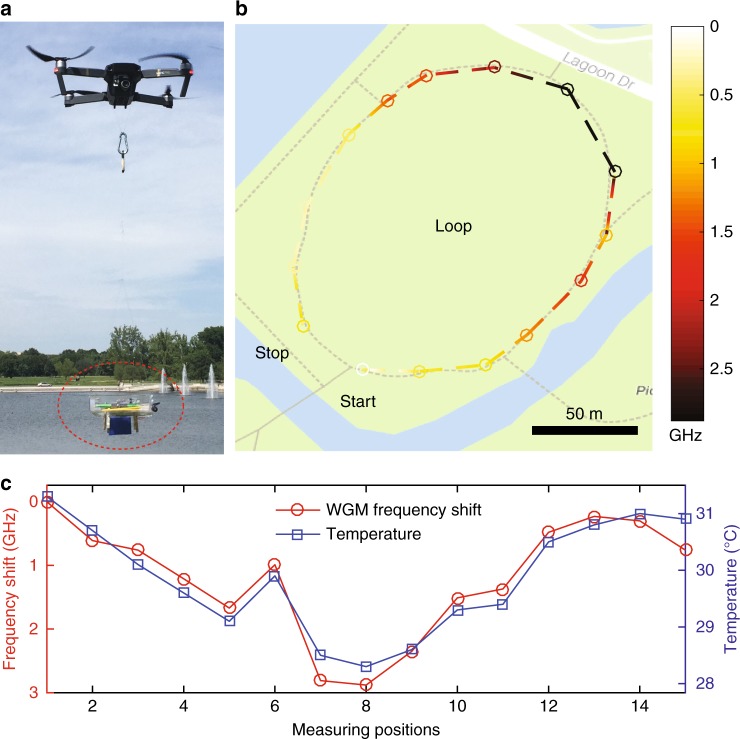
Aerial mapping of the temperature distribution. An unmanned drone was used to carry the wireless sensing system to measure the temperature distribution of a selected area in a city park of St. Louis. A commercial thermometer with a bluetooth connection was mounted together with the packaged sensor for comparison. **a** Photograph of an unmanned drone carrying the wireless sensing system (marked in the red dashed ellipse). **b** The frequency shift of the selected resonance mode when the drone flew in a selected loop; the starting and ending positions are marked. The resonance frequency at the starting position is set to be zero. The color bar represents the amount of frequency shift. The background image comes from Google Maps. Bar: 50 m. **c** Comparison of the measured frequency shift with the results from a commercial thermometer. The increasing numbers denote the positions of the measurements when the drone flew from the starting position to the ending position

In summary, we have demonstrated a wireless WGM sensor node and exploited it for the applications of thermal sensing and aerial mapping. A customized iOS app enables us to monitor and control the system parameters, as well as acquire and analyze the sensing signals in real time. Two application scenarios have been studied: one is measuring the temperature change at a fixed location; the other one is having the system carried by an unmanned drone to measure the temperature distribution of a selected area. Successful demonstrations show the potential applications of our wireless WGM sensor node in the IoT. Notably, our sensing system is not limited to thermal sensing. With the proper design, the packaged WGM sensor can have various functionalities, e.g., a WGM magnetometer with a high sensitivity and large bandwidth^18^.

In practical sensing scenarios, WGM optical sensors are often simultaneously subject to multiple stimuli, which affect both the accuracy and sensitivity of the measurement. Digital signal processing methods, e.g., maximum likelihood estimation, can contribute to extracting useful information from a noisy environment with a limited number of spectral sampling points^29^. Furthermore, through signal processing and learning approaches, we could assemble multiple WGM sensor nodes to form a sensor array to acquire more accurate results. The sensor array can also help to identify multiple environmental changes and stimuli simultaneously^30^.

## Electronic supplementary material

Supplementary Information

A video demo mentioned in the main text
