# Supplementary material for: Wireless whispering-gallery-mode sensor for thermal sensing and aerial mapping
Source: Light Sci Appl. 2018 Sep 12;7:62. doi: 10.1038/s41377-018-0063-4 (PMC6133935; doi:10.1038/s41377-018-0063-4)
Supplement: Supplementary file 1 — Supplementary Information [file 41377_2018_63_MOESM1_ESM.docx]

**Supplementary Information**

**Wireless whispering-gallery-mode sensor for thermal sensing and aerial mapping**

Xiangyi Xu^1^, Weijian Chen^1^, Guangming Zhao^1^, Yihang Li^1^, Chenyang Lu^2^ and Lan Yang^1^*

^1^Department of Electrical and Systems Engineering, Washington University, St. Louis, MO 63130, USA

^2^Department of Computer Science and Engineering, Washington University, St. Louis, MO 63130, USA

Correspondence: Lan Yang, Email: [yang@seas.wustl.edu](mailto:yang@seas.wustl.edu), Tel. No: +1(314)935-9543, Fax No: +1(314)935-7500.

**Instruction of the customized iOS App for the wireless WGM sensing system**

The customized iOS app “microCavity” can be downloaded in apple store with link <http://appsto.re/cn/c5lvib.i>. The main interface of microCavity is shown in **Fig. S1**. Its functions include data acquisition, chart display, system monitor, sensing measurement, and laser control. The tab interface allows users to monitor and control the electronic and optical systems, acquire and analyze the transmission spectrum of the packaged whispering-gallery-mode sensor.

**
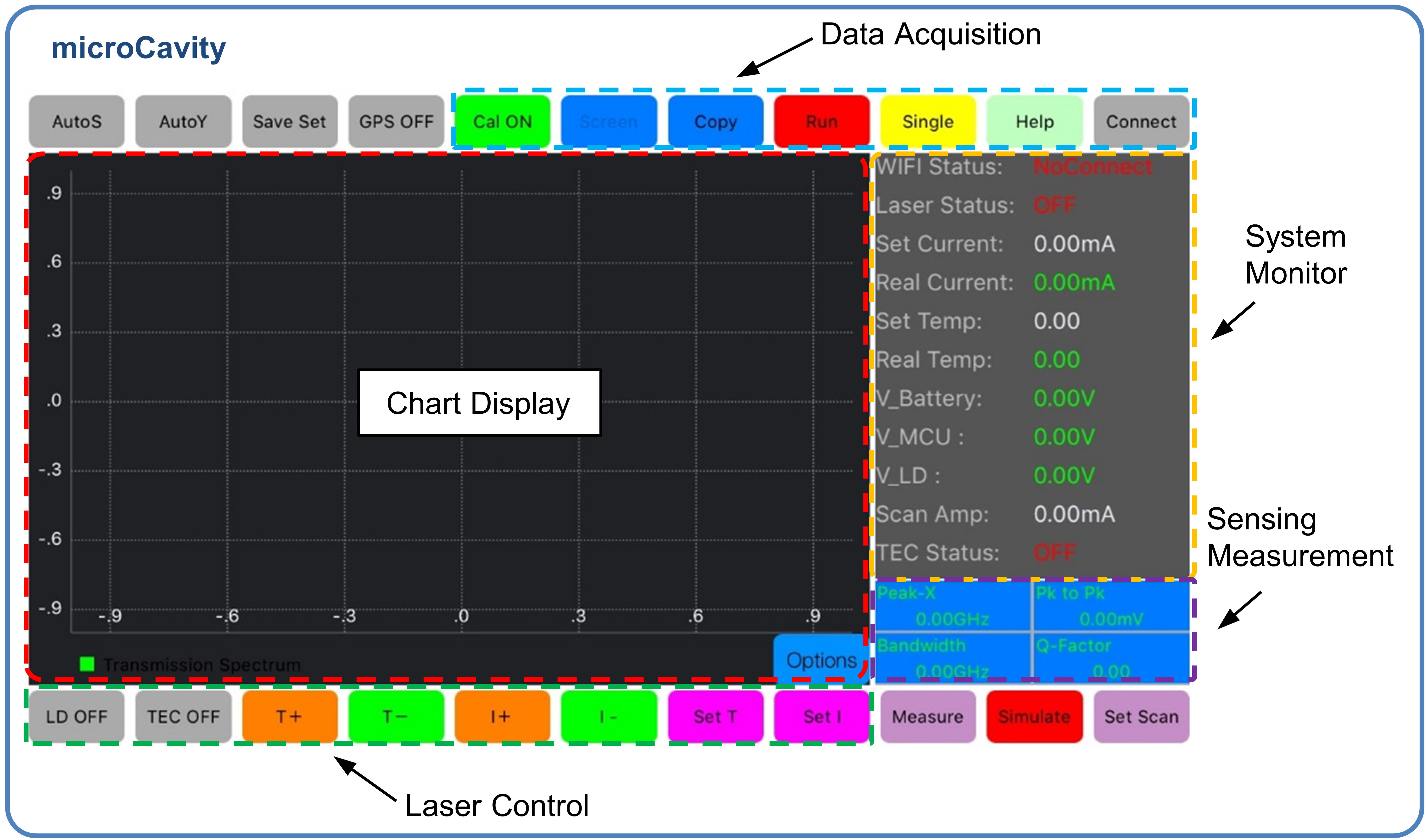
**

**Fig. S1 Screenshot of the customized iOS App for the wireless sensing system.** The main functions include data acquisition, chart display, system monitor, sensing measurement, and laser control.

Here we provide a step-by-step guide for this iOS app. The bold texts correspond to buttons in the app.

1. The first step is to connect the smartphone with the mainboard by clicking the **Connect** button. Once successfully connected, the Wi-Fi status will change from ‘*NoConnect*’ to ‘*Connected*’.
2. Then, pushing the **Run** button will enable the app to start acquiring data from the mainboard via the Wi-Fi module.
3. Under the laser control area, the current of the laser diode (I) and the TEC temperature (T) can be tuned to locate the target mode. Both T and I can be adjusted either step-by-step (**T+**, **T-**, **I+**, **I-**) or numerically (**Set T**, **Set I**).
4. After locating a resonance mode, typical parameters such as resonance frequency, linewidth, quality factor will be calculated and displayed in the front panel. By pushing **Measure** button, more parameters about the transmission spectrum will be displayed. In the **Option** menu, more options for spectrum display are available, which can help users to better visualize the transmission spectrum.
5. To export the data, users could use **Screen** button to copy the screenshot or **Copy** button to save the raw data to clipboard.

Below are the functions of the buttons not mentioned above.

**AutoS**, **AutoY**: sets the scale of both axes in the chart display. The scale can be either automatically adjusted or fixed.

**GPS**: provides the current location of the terminal.

**Cal**: provides calibration function and is used to clear out the background spectrum.

**Single**: allows users to acquire single transmission spectrum after pushing the button. The transmission spectrum won’t be updated until the next press. Note, the default mode of spectrum display is in real time enabled by pushing the **Run** button.

**Simulate**: is for debug or tutorial purpose and provides a typical Lorentzian spectrum that is integrated within this app.
